# Timeliness and completeness of monthly disease surveillance data reporting, Uganda, 2020-2021

**DOI:** 10.11604/pamj.2023.46.3.40557

**Published:** 2023-09-06

**Authors:** Robert Zavuga, Richard Migisha, Doreen Nsiimire Gonahasa, Daniel Kadobera, Benon Kwesiga, Paul Edward Okello, Lilian Bulage, Freda Loy Aceng, Joshua Kayiwa, Issa Makumbi, Alex Riolexus Ario

**Affiliations:** 1 Uganda Public Health Fellowship Program, Uganda National Institute of Public Health, Kampala, Uganda,; 2 Department of Integrated Epidemiology, Surveillance and Public Health Emergencies, Ministry of Health, Kampala, Uganda,; 3 National Public Health Emergency Operations Center, Uganda National Institute of Public Health, Kampala, Uganda

**Keywords:** Timeliness, completeness, monthly reports, Uganda

## Abstract

**Introduction:**

timely and complete reporting of routine public health information about diseases and public health events are important aspects of a robust surveillance system. Although data on the completeness and timeliness of monthly surveillance data are collected in the District Health Information System-2 (DHIS2), they have not been routinely analyzed. We assessed completeness and timeliness of monthly outpatient department (OPD) data, January 2020-December 2021.

**Methods:**

we analyzed secondary data from all the 15 regions and 146 districts of Uganda. Completeness was defined as the number of submitted reports divided by the number of expected reports. Timeliness was defined as the number of reports submitted by the deadline (15^th^ day of the following month) divided by reports received. Completeness or timeliness score of <80% was regarded incomplete or untimely.

**Results:**

overall, there was good general performance with the median completeness being high in 2020 (99.5%; IQR 97.8-100%) and 2021 (100%; IQR 98.7-100%), as was the median timeliness (2020; 82.8%, IQR 74.6-91.8%; 2021, 94.9%, IQR 86.5-99.1%). Kampala Region was the only region that consistently failed to reach ≥ 80% OPD timeliness (2020: 44%; 2021: 65%). Nakasongola was the only district that consistently performed poorly in the submission of timely reports in both years (2020: 54.4%, 2021: 58.3%).

**Conclusion:**

there was an overall good performance in the submission of complete and timely monthly OPD reports in most districts and regions in Uganda. There is a need to strengthen the good reporting practices exhibited and offer support to regions, districts, and health facilities with timeliness challenges.

## Introduction

Timely and complete reporting of routine public health information about diseases and public health events are important aspects of a robust surveillance system [1]. Through public health surveillance systems, information is continuously and systematically collected, analyzed, interpreted, and disseminated to guide the planning and implementation of public health programs [2]. Recurrent outbreaks which sometimes can lead to widespread epidemics and transmission to other countries demonstrate the need of having a surveillance system that provides complete information that allows it to detect changes in disease patterns in time to mount a response. Despite the increased efforts of strengthening health information reporting at different levels, low-income countries (LICs) are still challenged with untimely, incomplete, and inaccurate surveillance information which in turn affects the planning, monitoring, and evaluation of health sector performance and service delivery [3]. The introduction of a paperless system is one of the ways employed to improve the timeliness and completeness of reporting public health information and events in Uganda. The Ministry of Health (MoH) in Uganda operates a web-based information system known as the District Health Information System Version 2 (DHIS2) in which the data that are routinely generated from health facilities are filled in on a weekly and monthly basis. In this system, all the primary data received from lower-level health facilities which are captured in paper format are entered into the DHIS2 at the district level [3].

This interchange and transfer of data from paper into the DHIS2 likely caused distortions in terms of accuracy, timeliness, and completeness [4]. The DHIS2 has undergone three significant revisions and upgrades since its inception in 2010 and thus has the following versions: 2010-2014, 2015-2019, and 2020-2024. These revisions and upgrades were aimed at improving system performance and to also incorporate the new districts and regions that have been created from time to time. The Integrated disease surveillance and response (IDSR) indicators guide that for a report to be timely, 80% of health facilities must have submitted in time and for a report to be complete 80% of the expected reports should have been submitted [5]. A weekly epidemiological report published in January 2022 indicated that only 8 of the 15 regions in Uganda met the completeness target of 80% and no region met the timeliness target [6]. Subpar timeliness or completeness may lead to delayed detection of infectious diseases and the potential for larger outbreaks than would otherwise occur. Although data on the completeness and timeliness of surveillance data are collected in the DHIS2 2020-2024 version, they have not been routinely analyzed. The reporting of outpatient department (OPD) data in the years 2020 and 2021 could have been interrupted by the coronavirus disease 2019 (COVID-19) pandemic that was at the peak in this period [7], and therefore there could be some areas of weakness in the surveillance system. Thus, by periodically evaluating the timeliness and completeness of reporting of routine surveillance data, it is possible that specific barriers and challenges to reporting could be identified and immediately improved upon. Ultimately, improvement across these metrics should enable rapid and timely response to disease outbreaks and mounting of control measures. We estimated the timeliness and completeness of monthly OPD disease surveillance reports submitted to the DHIS2 in Uganda from January 2020-December 2021 to provide evidence-based recommendations to the MoH.

## Methods

**Study setting and design:** we conducted a descriptive quantitative study that involved analysis of monthly OPD disease surveillance reports submitted to the DHIS2 from January 2020 to December 2021. Uganda has 146 districts which are distributed across 15 regions as designated by MoH. These are; Acholi, Ankole, Bugisu, Bukedi, Bunyoro, Busoga, Kampala, Karamoja, Kigezi, Lango, North central, South Central, Teso, Tooro and West Nile. The health care system has several governments and privately owned health facilities which are organized in a hierarchical order [8]. At the bottom are the community health workers also known as the Village health team (VHT) members who report observations to the nearest health centers (HC). The lowest health centers are HCIIs (found at parish level), followed by HCIIIs (found at sub-county level), HCIVs (found at county/health sub-district level), general hospitals (found at district level), regional referral hospitals (found at regional level) and the national referral hospitals found at national level.

**Data source:** we extracted data from the DHIS2 from all the regions of Uganda which comprise the 146 districts. The DHIS2 is a web-based open-source health management information system used to collect aggregate data which is routinely generated across health facilities [9]. The DHIS2 also has capabilities for data analysis, data management, and data visualization. The DHIS2 automatically determines completeness and timeliness. The reports that are submitted by the deadline date are considered to be timely and the proportion of the actual number of reports submitted against the expected number of reports are regarded as complete. The monthly OPD reports are submitted in three different categories namely: nationals, refugees, and foreigners. For the purpose of this study, OPD reports from only nationals were considered.

**Generation of monthly surveillance data:** routine monthly surveillance data are generated at the community through surveillance activities carried out by the VHTs. The disease surveillance reporting system follows a hierarchical order from the community level to the national level through the DHIS2. At the health facility level, information is first collected as patient-specific data using paper-based IDSR surveillance tools and later transferred into the electronic format-the DHIS2 as aggregated data. The monthly OPD report is an aggregated report for all OPD occurrences at each health facility. It contains data on OPD attendances, referrals, diagnosis, infectious disease and epidemic prone diseases, non-communicable diseases, maternal and child health, family planning, and immunization services. The IDSR defines completeness as the proportion of reports submitted divided by the number of expected reports from the same health facility, district or region in a given time period while timeliness is defined as the proportion of reports submitted by the deadline divided by actual reports received in the given time period. Health facilities are expected to submit complete monthly reports by the 15^th^ of the following month. The facilities with percentages below the 80% target, are regarded as having submitted incomplete or untimely reports. Data from different health facilities are sent to the district and then later merged into regions constituting different districts.

**Data abstraction and analysis:** we captured different variables for the years 2020 and 2021. These years were considered because we wanted to evaluate the immediate past performance trends of the reporting indicators especially during the advent of COVID-19. A data abstraction form was used to extract information on the expected number of reports, actual number of reports and actual number of reports on time. It is from these variables that the monthly completeness and timeliness were computed. Completeness was calculated as the number of actual monthly OPD reports received divided by the expected number of reports in a given year and expressed as a percentage. Timeliness was calculated as the number of actual monthly OPD reports received on time (by the 15^th^ of every month) divided by the expected number of reports in a given year and was also expressed as a percentage. We determined the overall proportions of completeness and timeliness of reporting by year at national, regional, and district levels, level of health facility, and health facility ownership. We analyzed data using Epi-info version 7.0 (CDC, Atlanta, USA).

**Ethics approval and consent to participate:** because our study used routine surveillance data reported by health facilities in the DHIS2 which were also aggregated with no individual patient identifiers, we did not seek for ethical approval. However, we sought permission to use the data from the Uganda MoH. The US Centers for Disease Control and Prevention (CDC) also provided the non-research determination (NRD) for non-human subjects. Data were only accessed by the study team.

## Results

Overall, in 2020 the expected number of reports was 69,468 and of these, 68,935 reports were submitted and 52,430 were submitted in time corresponding to 99.2% completeness and 75.5% timeliness. In 2021, the expected number of reports of was 69,659 and of these 61,490 were submitted in time corresponding to 99.8% completeness and 88.1% timeliness. The median completeness of facility OPD reports was high in 2020 (99.5%; IQR 97.8-100%) and 2021 (100%; IQR 98.7-100%), as was the median timeliness (2020, 82.8%, IQR 74.6-91.8%; 2021, 94.9%, IQR 86.5-99.1%). There was a general improvement in reporting in terms of completeness and timeliness from 2020 to 2021. This trend is similar across all regions, level of health facilities, and type of ownership of health facility. Regarding completeness, all regions scored above the required reporting target of 80% in 2020 and 2021. However, 7 out of 15 regions did not reach the timeliness reporting target in 2020 and in 2021, only Kampala region did not score above the timeliness target. Kampala region was the only region which consistently failed to meet the 80% timeliness target in both years (2020: 44.4%; 2021: 64.7%). All levels of health facilities scored above the required completeness target in both years. National referral hospitals were the only facilities that consistently failed to meet the timeliness target in both years (2020: 47%; 2021: 74%). Privately owned health facilities failed to score above the required timeliness target in 2020. Although they improved and scored above the timeliness target in 2021, they still performed poorer than government owned facilities (Table 1).

**Table 1:** completeness and timeliness of monthly outpatient department reporting per region, level of health facility, and level of ownership, Uganda, 2020-2021

	2020		2021	
	Completeness (%)	Timeliness (%)	Completeness (%)	Timeliness (%)
**Region**				
Acholi	98.7	73.2	98.9	88.7
Ankole	99.8	77.1	100	89.9
Bugisu	98.3	84	100	96.9
Bukedi	97.1	82.5	96.8	86.3
Bunyoro	96.8	72.9	99.1	87.7
Busoga	98.6	74.1	99.9	86.1
Kampala	100	44.4	100	64.7
Karamoja	98.8	81.1	98.9	93.4
Kigezi	99.1	95.2	100	99.6
Lango	100	83.6	100	87.4
North central	100	70.1	100	88.2
South central	96.4	75.3	97.6	88.8
Teso	98.6	81.1	97.3	89.4
Tooro	98.4	81.4	99.4	95.5
West Nile	100	91.3	100	97.9
National level	99.2	75.5	99.8	88.1
**Level of health facility**				
Health center II	96.6	73.8	99.8	87.4
Health center IIII	98.7	80.0	99.1	90.9
Health center IV	97.9	79.7	99	89.9
District hospital	99.8	75.9	100	88.6
Regional referral	99.0	67.2	100	83.8
National referral	100	47.2	100	74.1
**Health facility ownership**				
Government	99.1	80.4	99.7	91.6
Private	100	69.9	100	84.2

**Figure 1:**
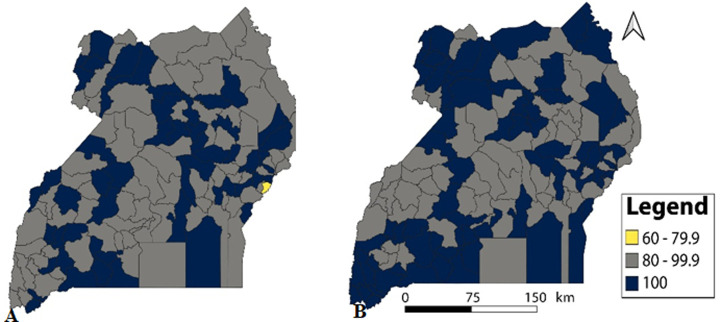
completeness of monthly outpatient department reports, Uganda, by district, A) 2020; B) 2021

**Figure 2:**
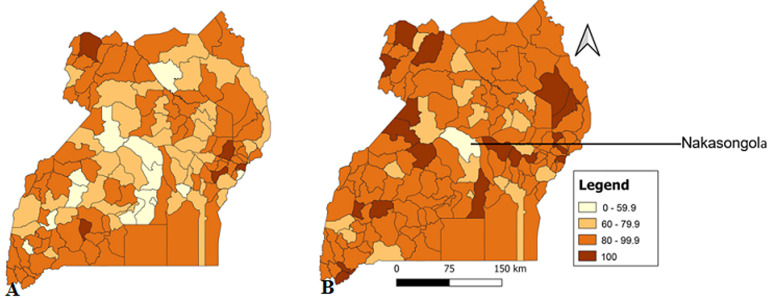
timeliness of monthly outpatient department reports, Uganda, by district, A) 2020; B) 2021

**District monthly completeness and timeliness of outpatient department reports, Uganda, 2020-2021:** in 2020, all districts scored above the recommended target for completeness except Namisindwa while in 2021, all districts scored above the required target (Figure 1). In regard to timeliness, 59 (40%) districts failed to submit monthly OPD reports on time in 2020 and 21 (14.4%) districts failed to submit on time in 2021. Nakasongola was the only district which consistently performed poorly in submission of timely reports by scoring below 80% in both years (2020: 54.4%, 2021: 58.3%) (Figure 2).

## Discussion

In our analysis of the monthly disease surveillance reporting data, we found that there was good performance in completeness and timeliness of reporting. In 2020, half of the regions did not submit reports in time. Kampala Region was the only region which consistently failed to reach the timeliness target in both years. At district level, Nakasongola District was the only district which consistently failed to score the timeliness target. Additionally, higher level health facilities like national referral hospitals and referral hospitals performed poorer than lower-level health facilities in terms of submission of timely reports. This study addresses the importance of monitoring routine surveillance data especially that which is collected on a monthly basis for public health action and monitoring performance. There was good performance in completeness and timeliness of reporting. This is due to regular mentorship and improved capacity of district biostatisticians and data personnel to collect and submit disease surveillance data [10]. Additionally, the switch from paper-based reporting to electronic internet based reporting and improved information and communication technology (ICT) capabilities has played a big role in improving performance of reporting disease surveillance data [11]. These observations are in line with other studies. A study which was done to explore the challenges in implementing surveillance tools of high income countries (HICs) and in low middle income countries (LMICs) indicates that improvement of capacity of health workers in data collection through education and mentorship improves the performance of surveillance information reporting [12]. Furthermore, a study which was done in Tanzania about use of technology innovations and ICT reported that improved ICT services provide an opportunity for better reporting and early detection of diseases [13]. In 2020, almost half of the regions did not achieve the 80% timeliness target. This was likely because of task shifting during response to the COVID-19 pandemic during this time; various health workers including medical data personnel were assigned other duties of active case finding and contact tracing and left the data departments understaffed thus affecting the timeliness of reporting [14]. Task shifting has been identified as an effective strategy in times of human resource scarcity. However, a Ugandan study, revealed that it may lead to low efficiency in performance of core functions and this affects quality of the tasks assigned [15].

On the other hand, a systematic review done on task shifting in sub-Saharan Africa recognized that although it is cost-effective, it has a risk of competing with other health service priorities [16]. Task shifting should be done without stifling the responsibilities of the mother departments. At district level, Nakasongola was the only district which consistently performed poorly in submission of timely reports in both years. This could be because Nakasongola is a district with several remote areas which have low information access [17]. Submission of untimely reporting has been linked to health facilities located in remote areas. A study done in the Solomon Islands revealed that health facilities located in remote areas had challenges in submitting timely reports [18]. Kampala Region did not reach the timeliness target in the period of two years. It is not certain why this region performed poorly, but it could be attributed to the large volume of patients it handles because of its big population and the corresponding patient flow. Kampala is the largest city in Uganda with a population of about 1.5 million people [19]. The high workload experienced by Kampala health facilities may have an impact on timeliness of reporting. High workload is known to affect timeliness and completeness of reporting. A study done in the Oceania Region in the Solomon Islands about malaria surveillance reporting system in the DHIS2 revealed that high workload leads to delays in timely reporting [18]. At health facility level, low timeliness was observed in higher health facilities like regional referral hospitals and national referral hospitals as compared to the lower health facilities. The reason for low timeliness in higher level health facilities in Uganda is not well documented. However, higher level health facilities have more service points and generate more data than lower level health facilities [20]. The data generated is mainly in the paper-based format. The process of collection and collation of this huge amount of data from the paper-based system into the electronic system could lead to the delays in timely submission. It is therefore important that primary data entry is electronically done to avoid delays.

**Limitation:** our study only utilized DHIS2 information in the years of 2020 and 2021. We did not analyze reporting rates for a longer duration of time that is, the years before 2020. This could have allowed us ascertain the true reporting trends over the years. We intended to analyze reporting rates during the COVID-19 pandemic to ascertain if surveillance data reporting was affected since the health system was affected during this period. Our study has a strength that it utilized disease surveillance data which were collected from the whole country.

## Conclusion

There was good reporting in terms of completeness and timeliness in both years. However, despite the good reporting, timeliness of reporting was low in Nakasongola District, Kampala Region and higher-level health facilities. We recommended strengthening the practices leading to good reporting and offer support to health facilities with challenges to timeliness through mentorships and continuous support supervision. Further studies are needed to understand and identify barriers to timely reporting.

### What is known about this topic

- *Timely and complete reporting is an important aspect in disease surveillance as it helps in timely detection of disease outbreaks and public health events;*
- *There are challenges faced by low resource settings especially in Africa in submission of timely and complete disease surveillance data and later on its utilization;*
- *The integrated disease surveillance and response guidelines currently recommend that for a report to be timely, 80% of the reporting units must have submitted in time and for a report to be complete 80% of the expected reports should have been submitted*.

### What this study adds

- *The study demonstrates the need for periodic analysis of routinely collected surveillance data to facilitate planning and decision-making;*
- *The results show that there was good performance in submission of monthly diseases surveillance data, this is in contrast to other studies which looked at submission of weekly reports where there was poor performance;*
- *The study opens an opportunity for future studies that need to investigate why there is good performance in submission of monthly reports yet there is poor performance in submission of weekly reports*.
